# Postnatal growth and childhood asthma in preterm and full‐term twins: A cohort study

**DOI:** 10.1111/pai.70488

**Published:** 2026-09-16

**Authors:** Resthie R. Putri, Cecilia Lundholm, Mwenya Mubanga, Christian Trillkott, Vilhelmina Ullemar, Lars Sävendahl, Catarina Almqvist

**Affiliations:** ^1^ Department of Medical Epidemiology and Biostatistics Karolinska Institutet Stockholm Sweden; ^2^ Strategic Information Unit The Centre for Infectious Disease Research in Zambia Lusaka Zambia; ^3^ Department of Oncology‐Pathology Karolinska Institutet Stockholm Sweden; ^4^ Department of Gynecology and Reproductive Medicine Karolinska University Hospital Stockholm Sweden; ^5^ Division of Pediatric Endocrinology, Department of Women's and Children's Health (KBH) Karolinska Institutet Stockholm Sweden; ^6^ Pediatric Endocrinology Unit Karolinska University Hospital Stockholm Sweden; ^7^ Pediatric Allergy and Pulmonology Unit Astrid Lindgren Children's Hospital, Karolinska University Hospital Stockholm Sweden

**Keywords:** asthma, postnatal growth, preterm birth, twins

## Abstract

**Background:**

The role of postnatal growth in asthma development in children remains unclear. We examined the association between postnatal growth pattern and childhood asthma and assessed whether this association differed between preterm and full‐term children.

**Methods:**

This cohort study included twins from the Child and Adolescent Twin Study in Sweden (born 1992–2003), linked to national registers. Postnatal weight and height to age 5 were obtained from child health records and standardized for age and sex (SDS). Childhood asthma was defined by parent‐reported asthma at age 9 or 12. Analyses were stratified by preterm (<37 weeks) and full‐term birth.

**Results:**

Among 3059 children, 15.6% developed asthma, 38.3% were born preterm. On average, the asthma group had 0.16 lower weight and 0.27 lower height SDS during the first 6 months, with no growth differences between asthma and non‐asthma groups thereafter. These early differences were apparent among preterm children. From the identified growth trajectories, normal birth size with subsequent normal growth occurred in 79.1% of full‐term and 41.9% of preterm children. A group with low birth size followed by catch‐up growth was associated with an increased odds of asthma (adjusted OR: 1.68, 95% CI: 1.16–2.45).

**Conclusions:**

Postnatal growth differences between children with and without asthma were limited to the first months of life among those born preterm, with similar growth patterns thereafter up to age 5 and among full‐term children. Among the identified growth trajectories, childhood asthma was most prevalent in those with low birth size and subsequent catch‐up growth.

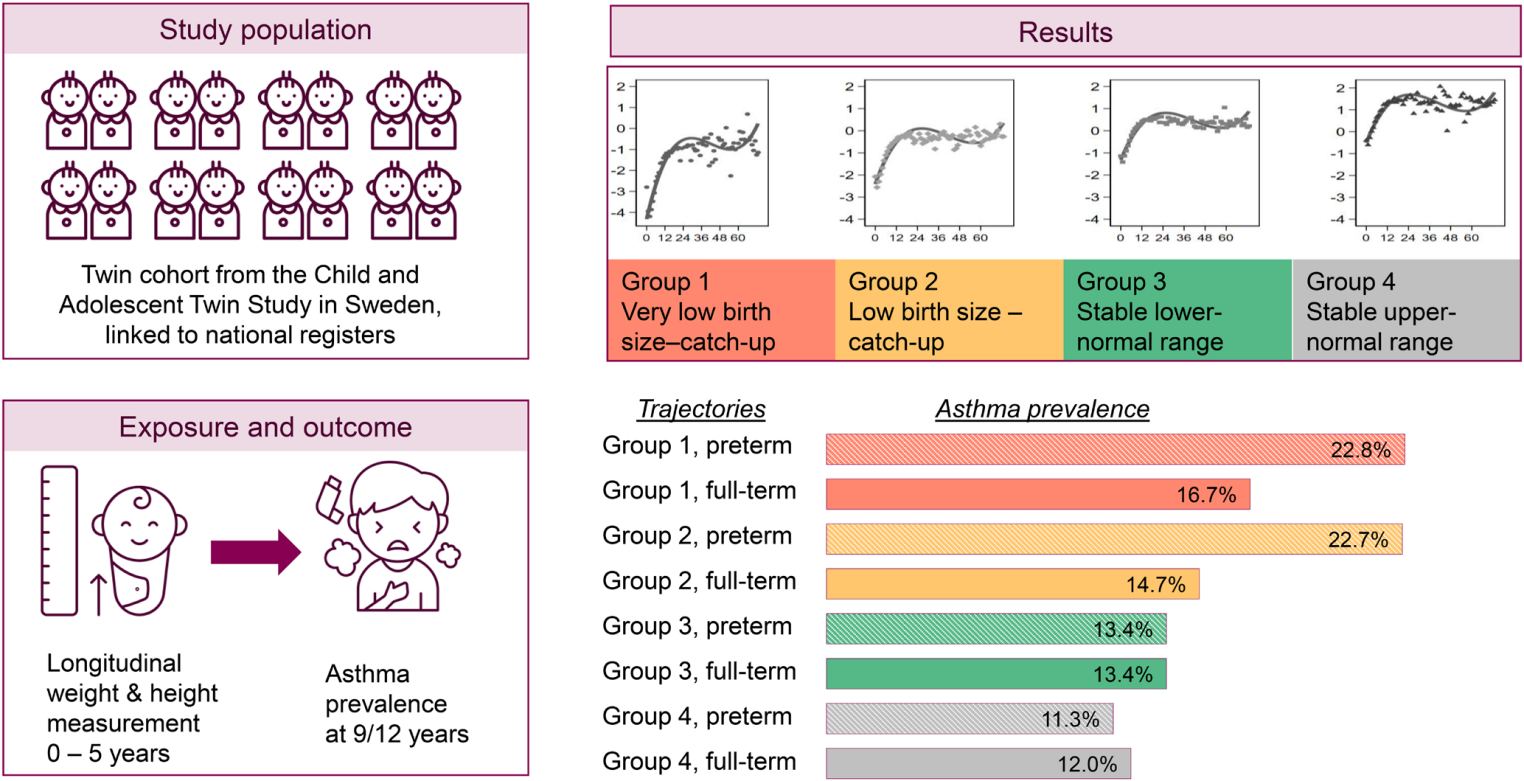

## Graphical abstract

In this twin cohort study, increased risk of asthma was observed in children with low birth size followed by catch‐up growth.

**Key message**

In this twin study, asthma‐related growth differences were observed only during early infancy among preterm children, with no differences thereafter, and none among full‐term children. Low birth size followed by catch‐up growth was associated with an increased risk of childhood asthma. These findings help clarify the association between early childhood growth and asthma and highlight differences between preterm and full‐term children.

## 1 INTRODUCTION

Asthma remains the most common chronic disease in children.^1^ Early‐life growth is a key determinant of lung development,^2^ yet its association with childhood asthma is complex. Impaired fetal growth has consistently been associated with an increased risk of childhood asthma in previous literature, including meta‐analyses.^3^, ^4^ In contrast, the role of postnatal growth in asthma remains inconclusive despite extensive literature. Weight gain, but not height gain, has been associated with airway dysanapsis,^5^, ^6^ potentially contributing to asthma, while persistently low growth in childhood appears to be associated with reduced lung growth.^7^ Further, studies of postnatal growth and asthma have rarely assessed whether gestational age at birth modifies this association,^8^ or have been restricted to full‐term populations.^4^, ^9^ Consequently, it remains unclear whether postnatal growth influences asthma risk differently in preterm and full‐term children. Evidence on growth patterns and subsequent respiratory outcomes among preterm children remains limited.^10^

Twin studies provide a valuable framework to disentangle genetic and environmental contributions to growth and disease, but twins are often excluded from asthma research due to earlier birth and smaller size, despite such differences not necessarily limiting generalizability.^11^ Using a nationwide twin cohort, we aimed to investigate associations between postnatal growth, assessed as average growth and growth trajectories, and asthma, and whether these associations differ between children born preterm and full‐term. We hypothesized that postnatal growth differs between children with and without asthma.

## 2 METHODS

### 2.1 Study design and population

A cohort study of twin children enrolled in the Child and Adolescent Twin Study in Sweden (CATSS), linked with health and sociodemographic national registers, was conducted. CATSS is an ongoing population‐based longitudinal study of twins born in Sweden.^12^, ^13^ Since 2004, the Swedish Twin Registry has invited parents of twins to participate in CATSS, contacting them at age 9 years in most years and at age 12 years during the first 3 years of data collection.^12^, ^13^

**FIGURE 1:**
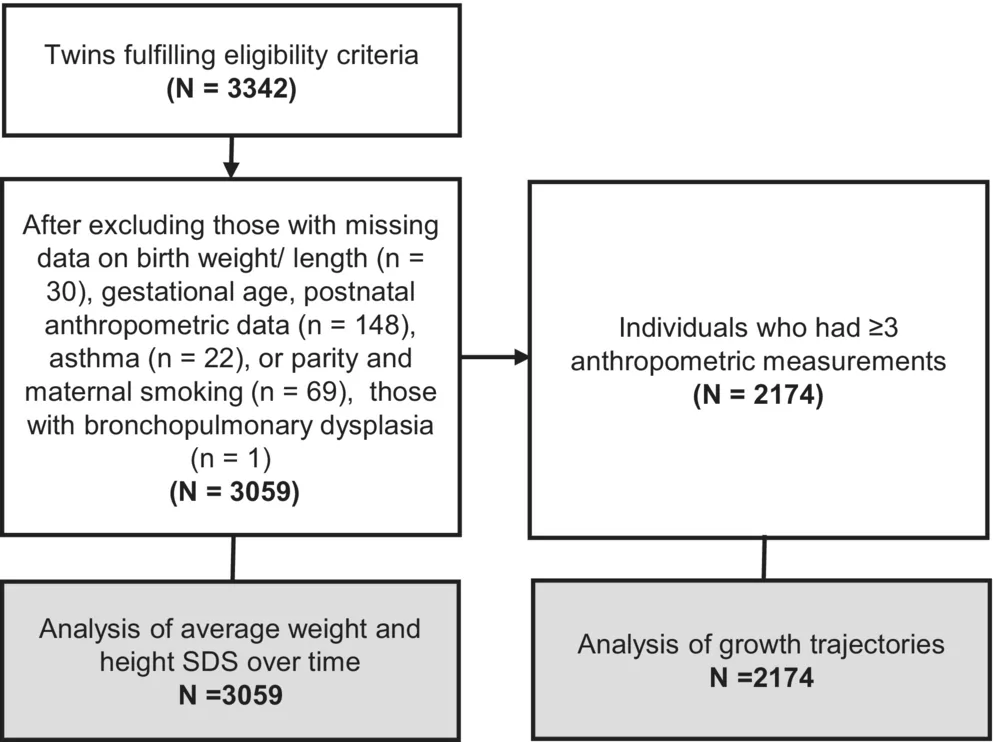
Flowchart of the study exclusion process.

Inclusion criteria were twins in CATSS born between 1992 and 2003, with consent obtained for retrospective collection of child health record data. Exclusion criteria included diagnosis of bronchopulmonary dysplasia and missing data on any of the following variables: birth weight or height, gestational age, postnatal weight or height, childhood asthma, parity, or maternal smoking data. The details of the exclusion process are shown as a flowchart in Figure 1. The Regional Ethical Review Board in Stockholm (Dnr 2018/1697‐31/1; 2023‐03916‐02) approved the study. As the study was based on linked register data, informed consent was waived in accordance with Swedish regulation and ethical review procedures.

### 2.2 Variables

The dependent variable, asthma (yes/no), was defined as positive responses to either of the International Study of Asthma and Allergies in Childhood questions “*Has your child ever had wheezing or whistling in the chest at any time?*” or “*Has your child ever had asthma?*”, together with a positive response to the question “*Was asthma diagnosed by a doctor or nurse?*” Information was obtained from parental interviews conducted when the twins were aged 9 or 12 years. Thus, the variable reflects ever having asthma up to the time of data collection, rather than current asthma.

Anthropometric measures at birth included birth weight (grams) and birth length (centimeters). Postnatal growth was assessed using repeated measurements of weight (kg) and height (cm) collected from birth until 71 months of age during routine health checkups at child health centers. Weight and height were standardized for age and sex according to the World Health Organization growth reference and expressed as standard deviation scores (SDS).^14^

Gestational age at birth was defined in completed weeks, based primarily on ultrasound estimates or, when unavailable, on last menstrual period or clinical birth records. Preterm birth (yes/no) was defined as delivery before 37 completed gestational weeks.

Several variables were considered as potential confounders. Maternal characteristics included age at delivery, parity, body mass index (BMI), history of asthma (yes/no), and smoking habit reported at antenatal care (yes/no). Parental education was defined as the highest educational level attained by either parent in the year of the child's birth and categorized as compulsory school (≤9 years), high school (10–12 years), or university level (>12 years).

Bronchopulmonary dysplasia, as an exclusion criterion, was identified using the International Classification of Diseases, 10th revision (ICD‐10) code P27.1.

### 2.3 Data sources

Asthma status and information on birth weight and length were obtained from the CATSS cohort,^12^, ^13^ with birth data cross‐checked and supplemented using the Swedish Medical Birth Register.^15^ Postnatal growth data, including measurements of weight, height, and dates of assessment, were extracted from health records from primary child health centers. Details on the collection of child health records are published elsewhere.^16^ When the measurement date was missing, an estimated date was derived based on the plotted growth curve. In Sweden, children undergo intensive health check‐ups from birth to 18 months of age, followed by an additional three to five assessments during the preschool years.^17^ Gestational age and maternal characteristics were obtained from the Medical Birth Register. Information on parental education was retrieved from the Longitudinal Integrated Database for Health Insurance and Labor Market Studies.^18^ Bronchopulmonary dysplasia was identified using data from the National Patient Register.^19^, ^20^ Detailed descriptions of each data source and the validity of the data are provided in Table S1.

### 2.4 Statistical analysis

In descriptive analyses, categorical variables are presented in numbers and proportions, whereas continuous variables are in median, first quartile (Q1), and third quartile (Q3). Individual‐level longitudinal data of weight and height SDS from 0 to 71 months were visualized using a scatterplot to assess nonlinearity.

We compared average weight and height growth from 0 to 71 months in children with and without asthma using generalized estimating equations (GEE), specifying a Gaussian family and an identity link function. Weight SDS and height SDS over time were used as separate dependent variables, with ever asthma as the main independent variable. Age was modeled as a continuous variable in days using restricted cubic splines with four knots, and an interaction term between asthma status and age was included to allow growth trajectories to differ by asthma status. An exchangeable working correlation structure was specified to account for within‐individual repeated measurements. Clustering due to twin pairs was accounted for by robust standard errors at the twin‐pair level. The unadjusted model included age and the interaction between age and asthma. The adjusted model, which constitutes the main analysis, additionally included maternal age at delivery, parity, maternal smoking during pregnancy, maternal asthma, and parental education. In the subset of children with available data on maternal BMI, a further model was fitted with additional adjustment for maternal BMI. Analyses were first conducted in the full population. Effect modification by preterm birth was assessed by including interaction terms between asthma, age, and preterm birth in the models. Analyses were then stratified by term and preterm birth. Adjusted means and the 95% confidence intervals (CI) were obtained from the GEE models using marginal predictions with covariates held at their sample means.

While population average models, such as GEE, describe overall growth patterns, individual trajectories may differ substantially. To characterize postnatal growth heterogeneity, we identified distinct growth trajectories using group‐based multi‐trajectory modeling,^21^ including individuals with weight and height data at ≥3 time points (Figure 1). Trajectory groups for weight and height SDS over time (in months) were identified. The Bayesian information criterion was used to guide the selection of the number of groups and the trajectory shape (third‐degree polynomial). Model adequacy was assessed according to the following criteria:^22^ (a) concordance between estimated group membership probabilities and the proportions assigned based on posterior probabilities; (b) average posterior probabilities >0.7; (c) odds of correct classification >5; (d) reasonably narrow confidence intervals for group membership probabilities; and (e) clinical relevance. Associations between growth trajectory groups and asthma were examined using logistic regression with cluster‐robust standard errors to account for clustering within twin pairs, and associations across combinations of preterm birth and growth trajectories were also assessed. To account for shared genetic and familial environmental factors, conditional logistic regression was conducted within twin pairs discordant for asthma. Odds ratios (ORs) and 95% confidence intervals (CIs) were reported. In all analyses, a two‐sided 5% significance level was applied, and analyses were conducted in Stata 18.

Microsoft Copilot (Microsoft Corp.) was used to check and improve readability and language clarity. No generative artificial intelligence was used to generate tables, figures, or original scientific content.

## 3 RESULTS

Of 3342 individuals, 3059 were included in the main analysis, Figure 1. Excluded individuals had a similar sex distribution but were born at a lower gestational age (50.4% female; median gestational age: 34 weeks). Among those included, 15.6% developed asthma, and 38.3% were born preterm. Characteristics of the study population are presented in Table 1.

**TABLE 1:** Characteristics of the study population.

	Total (*N* = 3059)	No asthma (*N* = 2582)	Asthma (*N* = 477)
Females, *n* (%)	1612 (52.7)	1422 (55.1)	190 (39.8)
Birth weight (gram), median (Q1, Q3)	2700 (2315, 3025)	2700 (2330, 3030)	2680 (2175, 2985)
Birth length (cm), median (Q1, Q3)	47 (45, 49)	47 (46, 49)	47 (45, 49)
Gestational age (weeks), median (Q1, Q3)	37 (35, 38)	37 (36, 38)	37 (35, 38)
Preterm birth, *n* (%)	1172 (38.3)	962 (37.3)	210 (44.0)
Maternal asthma, *n* (%)	121 (4.0)	88 (3.4)	33 (6.9)
Smoke exposure in pregnancy, *n* (%)	413 (13.5)	328 (12.7)	85 (17.8)
Maternal BMI, *n* (%)^a^	23.2 (21.3, 25.8)	23.1 (21.2, 25.8)	23.6 (21.7, 26.2)
Parental education, *n* (%)			
Compulsory school	196 (6.4)	149 (5.8)	47 (9.8)
High school	1493 (48.8)	1243 (48.1)	250 (52.4)
University	1370 (44.8)	1190 (46.1)	180 (37.8)

Abbreviations: BMI, body mass index; cm, centimeters; kg, kilograms; Q1, first quartile; Q3, third quartile.

^a^ Available for 2534 individuals (non asthma group, *n* = 2124: asthma group, *n* = 410).

### 3.1 Average longitudinal postnatal growth and childhood asthma

Scatterplots showing descriptive weight and height SDS over time are presented in Figure S1. The number of individuals contributing to the data at each age is shown in Table S2. The median number of anthropometric measurements per child was 15 (Q1: 4, Q3: 19).

**FIGURE 2:**
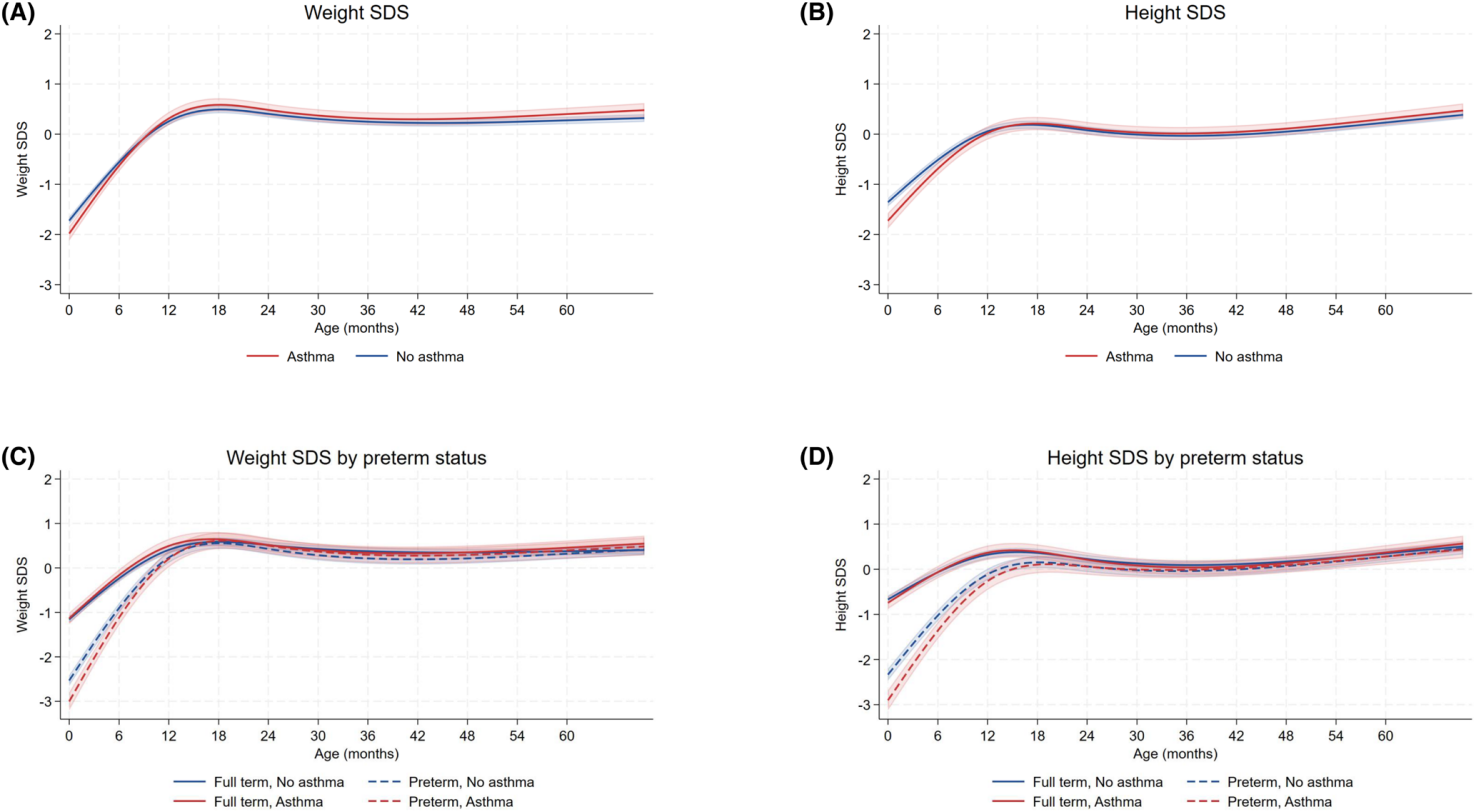
Mean weight and height SDS from birth to 71 months in children with and without asthma. Panels show: (A) adjusted weight SDS over time, (B) adjusted height SDS over time, (C) adjusted weight SDS over time stratified by preterm birth, and (D) adjusted height SDS over time stratified by preterm birth. SDS, standard deviation score. Average weight and height growth from birth to 71 months among children with and without asthma using generalized estimating equations (GEE) with a Gaussian family and an identity link was performed. Weight SDS and height SDS were modeled as separate dependent variables, with asthma status as the primary independent variable. An exchangeable correlation structure was applied and accounted for within‐twin correlation. The models included age in days (modeled using a restricted cubic spline), an interaction term between asthma status and age, maternal age, maternal smoking during pregnancy, maternal asthma, parity, and parental education. The estimated means for the adjusted analyses were performed with all covariates at means (i.e., mother's age at 30 years, no maternal asthma, no smoking, parental education at high school level, parity = 2).

In the overall study population, during the first 6 months, children in the asthma group had lower weight and height than those in the non‐asthma group, with adjusted mean differences across this period of approximately −0.16 SDS in weight and −0.27 SDS in height. Beyond this early period, no further differences between the asthma and non‐asthma groups were observed through the end of follow‐up (Figure 2A,B). Effect modification by preterm birth was observed for the association between asthma and weight SDS (*p* for interaction <0.001) and height SDS (*p* for interaction = 0.002).

When analyses were stratified by preterm birth, differences in early growth between asthma and non‐asthma groups were observed only among children born preterm, with no significant differences among those born full term (Figure 2C,D). Among preterm children, the adjusted mean weight SDS at birth was −3.00 in the asthma group compared with −2.53 in the non‐asthma group, and this gap persisted until approximately 3 months of age (−2.04 vs. −1.70 at 3 months). For height SDS, adjusted means were −2.87 versus −2.30 at birth, with significant differences remaining up to around 5 months. Among full‐term children, weight and height SDS were similar between the asthma and non‐asthma groups throughout follow‐up (e.g., at 2 months: weight SDS –0.71 vs. −0.75; height SDS –0.51 vs. −0.46). These patterns were similar in the unadjusted analyses (Figures S2 and S3) and in the analyses further adjusted for maternal BMI (Figure S4).

### 3.2 Postnatal growth trajectories and childhood asthma

**FIGURE 3:**
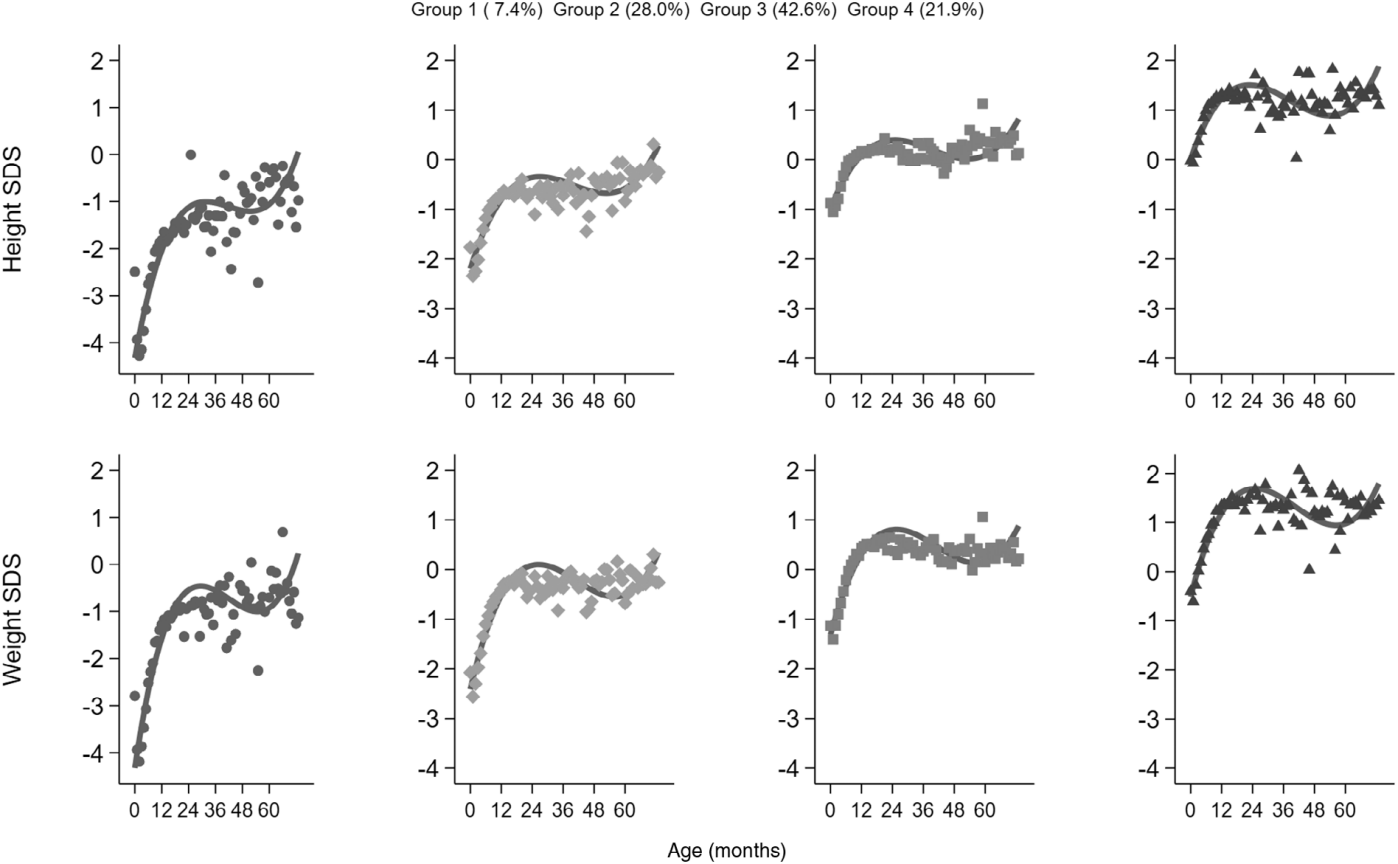
Four distinct postnatal weight and height trajectory groups. Each trajectory group was defined by weight SDS and height SDS using group‐based multi‐trajectory modeling. Based on the selection procedure,^22^ a four‐group model emerged as the preferred model.

Of those included for trajectory analysis (*N* = 2174), four distinct trajectory groups for weight and height SDS were identified (Figure 3). Excluded individuals had a similar sex and gestational age distribution (51.8% females; median gestational age: 37 weeks) to those included in the analysis.

*Group 1* (*7.4% of the study population*): Average weight and height SDS were below −4 at birth and increased over time, exceeding −2 SDS sometime between 1 and 2 years of age.

*Group 2* (*28.0%*): Average weight and height SDS were slightly below −2 at birth and exceeded −2 SDS before 1 year of age.

*Group 3* (*42.6%*): Average weight and height SDS started at around −1 SDS and remained around 0 SDS throughout.

*Group 4* (*21.9%*): Average weight and height SDS started at around 0 SDS and were consistently in the normal upper range.

**FIGURE 4:**
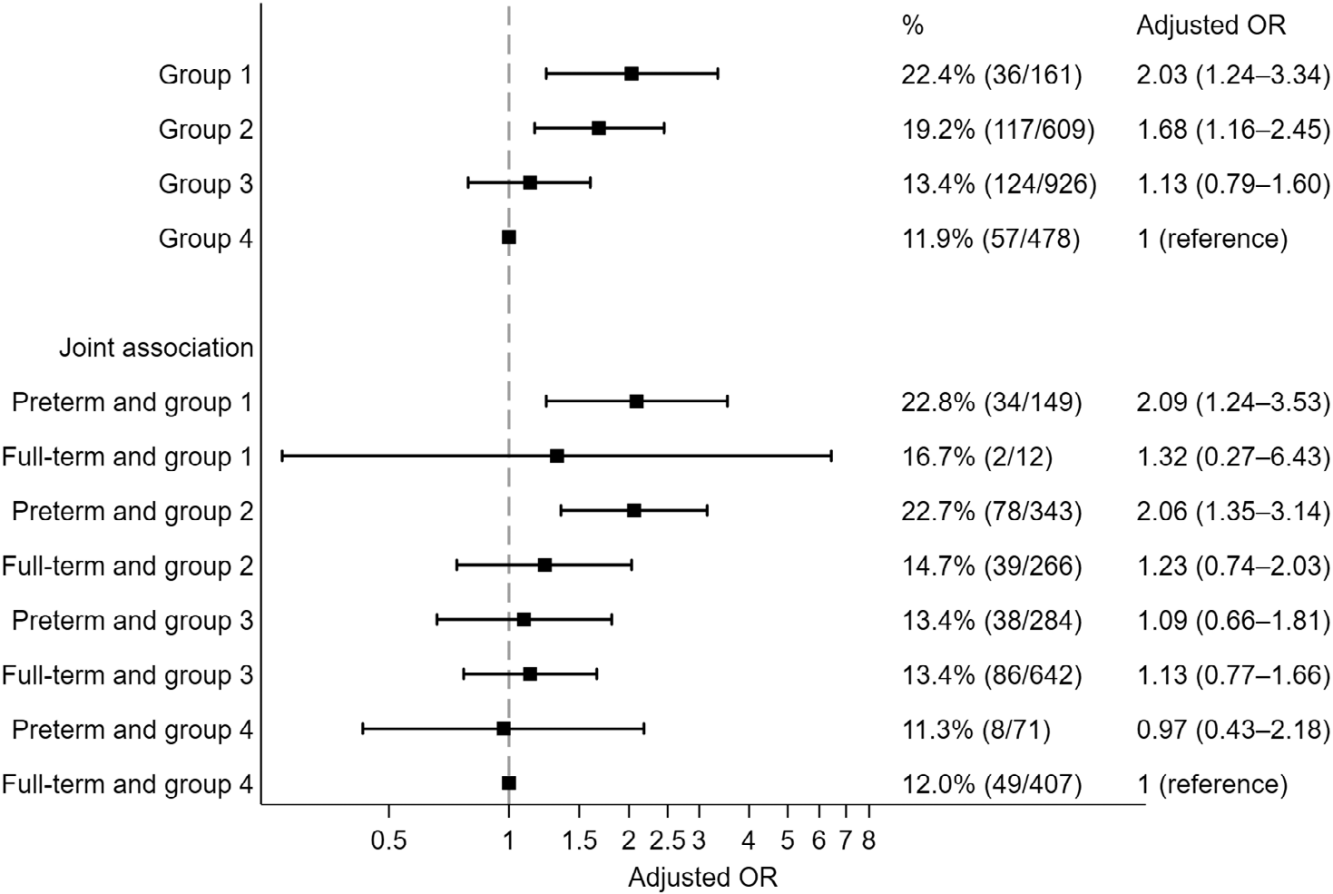
Association between postnatal growth trajectories and asthma. CI, confidence interval; OR, odds ratio. ORs and 95% CIs were estimated using logistic regression adjusted for maternal age, maternal asthma, smoking exposure during pregnancy, parity, and parental education.

Table 2 shows the characteristics of each trajectory group. As expected, the highest proportion of preterm birth was observed in group 1 (92.6%) and the lowest was in group 4 (14.9%). Similarly, the highest prevalence of asthma was found in group 1 (22.4%) and the lowest in group 4 (11.9%). Compared with trajectory group 4, the odds of asthma were higher in the two lowest trajectory groups: adjusted OR for group 2 was 1.68 (95% CI: 1.16–2.45) and for group 1 was 2.03 (95% CI: 1.24–3.34), independent of maternal age, maternal smoking, maternal asthma, parity, and parental education. In contrast, no significant difference was observed for trajectory group 3 (adjusted OR 1.13, 95% CI: 0.79–1.60), Figure 4. Similar results were observed after further adjustment for maternal BMI (Table S3).

**TABLE 2:** Comparison of demographic and clinical characteristics across the trajectory groups.

	Group 1	Group 2	Group 3	Group 4
	*N* = 161	*N* = 609	*N* = 926	*N* = 478
Females, *n* (%)	73 (45.3)	321 (52.7)	507 (54.7)	254 (53.1)
Birth weight (gram), median (Q1, Q3)	1590 (1253, 1860)	2275 (1990, 2590)	2788 (2510, 3015)	3078 (2860, 3375)
Birth length (cm), median (Q1, Q3)	41 (38, 43)	45 (44, 47)	48 (46, 49)	50 (48, 51)
Gestational age (weeks), median (Q1, Q3)	33 (31, 35)	36 (34,38)	38 (37, 39)	38 (37, 40)
Preterm birth, *n* (%)	149 (92.6)	343 (56.3)	284 (30.7)	71 (14.9)
Maternal asthma, *n* (%)	8 (4.8)	31 (5.1)	31 (3.4)	12 (2.5)
Smoking in pregnancy, *n* (%)	30 (18.6)	104 (17.1)	114 (12.3)	51 (10.7)
Maternal BMI, median (Q1, Q3)^a^	22.6 (21.3, 25.8)	22.9 (21.1, 25.9)	23.1 (21.2, 25.8)	23.7 (21.6, 25.8)
Parental education, *n* (%)				
Compulsory school	13 (8.2)	42 (7.0)	46 (5.0)	19 (4.0)
High school	87 (55.1)	310 (51.8)	419 (45.7)	207 (43.0)
University	58 (36.7)	246 (41.2)	452 (49.3)	255 (53.0)
Asthma, *n* (%)	36 (22.4)	117 (19.2)	124 (13.4)	57 (11.9)

Abbreviations: BMI, body mass index; cm, centimeters; kg, kilograms; m, meters; Q1, first quartile; Q3, third quartile.

^a^ Data on maternal BMI were available for 1793 individuals.

When associations by joint categories of preterm birth and growth trajectories were assessed (Figure 4), children born preterm and classified in trajectory groups 1 and 2 had the highest odds of asthma. Compared with full‐term children in trajectory group 4 (reference), preterm children in group 1 showed two‐fold increased odds of asthma (adjusted OR: 2.09, 95% CI: 1.24–3.53), and those in group 2 had similarly elevated odds (adjusted OR: 2.06, 95% CI: 1.35–3.14), whereas preterm children in groups 3 and 4 did not show significantly increased odds of asthma. Among full‐term children, no significant associations between trajectory groups and asthma were observed. The results were similar in unadjusted analysis and in analysis further adjusted for maternal BMI (Table S4).

Post‐hoc comparisons further clarified the observed pattern of associations. When comparing odds of asthma in preterm and full‐term children within each trajectory group, a significant difference was observed only in trajectory group 2 (adjusted OR 1.81, 95% CI: 1.27–2.60), with no significant differences in the other trajectory groups. When comparing odds of asthma between trajectory groups within preterm and full‐term children, differences were observed only among preterm children: those in trajectory group 3 had lower odds of asthma than those in trajectory group 2 (adjusted OR 0.53, 95% CI: 0.34–0.84). No corresponding differences were observed among full‐term children.

Of the 880 twin pairs included in the trajectory modeling, 163 were discordant for asthma, and among those, 69 pairs were also discordant for postnatal growth trajectories. Point estimates of the association between postnatal growth trajectories and asthma from the co‐twin control analyses were generally similar, but no longer statistically significant. Compared to group 4, the OR for group 1 was 2.08 (95% CI: 0.51–8.55); for group 2 was 1.28 (95% CI: 0.50–3.27); and for group 3 was 1.39 (95% CI: 0.63–3.04).

## 4 DISCUSSION

In our study population, early growth patterns differed between children with and without asthma only among those born preterm. Preterm children who later developed asthma had lower weight and height during the first 6 months of life, after which their weight and height SDS became similar to those without asthma. Among full‐term children, longitudinal growth from birth to 71 months did not differ between the asthma and non‐asthma groups. These findings suggest that differences in average growth associated with asthma among preterm children are limited to the first months of life, with overall growth patterns thereafter being similar regardless of asthma status.

Furthermore, our findings indicate substantial heterogeneity in postnatal growth, with the highest prevalence of asthma observed in children with the lowest birth size who experienced catch‐up growth to population‐average levels sometime between 1 and 2 years of age. This group had a lower gestational age at birth than the other trajectory group, suggesting that very preterm birth may drive this trajectory pattern. Further, when the results were stratified by gestational age, asthma risk across trajectory groups appeared similar among full‐term children, whereas the risk did not seem uniform among preterm children. The observed association between trajectory groups and asthma may be attributable to adverse fetal growth and prematurity, rather than solely to postnatal growth.

Looking into trajectory groups with low birth size (i.e., trajectory group 1 and group 2), interestingly, despite substantial differences in birth size, these groups showed similar asthma risk. Both groups exhibited catch‐up, although at different rates, and attained weight and height SDS above −2 during early childhood, while their trajectories remained largely below the population average during follow‐up. Nevertheless, because both trajectory groups 1 and 2 showed some degree of catch‐up growth, the independent effect of catch‐up growth could not be clearly assessed.

The co‐twin control analysis performed in this study yielded non‐significant associations with wide confidence intervals, reflecting limited statistical power. However, the point estimates were broadly similar to those from the full‐cohort analysis, suggesting that shared familial environmental and genetic factors alone may not fully explain the association between trajectory groups and asthma.

Our findings are broadly consistent with prior evidence. A meta‐analysis including 147,000 European children demonstrated a joint effect of younger gestational age and higher infant weight gain on increased asthma risk,^4^ aligning with our observation, while our results further add that growth beyond infancy may not be associated with childhood asthma. Moreover, consistent with our findings, a longitudinal study of 12,000 children born full‐term reported no clear association between growth in early childhood and asthma at 6 years of age.^23^

When comparing the preterm and full‐term children, a cohort study of offspring of mothers with high‐risk asthma reported that weight gain from 0 to 6 years was associated with a lower asthma risk among preterm children but a higher risk among those born full term.^24^ While we similarly observed that associations between growth and asthma varied by gestational age, this specific pattern was not evident in our study. These differences may partly reflect methodological variation, as the previous study assumed linear weight gain, whereas our approach captures the inherently non‐linear nature of postnatal growth,^25^ as well as differences in study populations (singleton‐only cohorts compared with our twin population).

Both lower gestational age at birth and greater infant weight gain are each associated with airflow obstruction measured by forced expiratory volume in 1 s (FEV_1_) and its ratio with force vital capacity (FEV_1_/FVC).^5^, ^26^ Moreover, the association between higher weight gain and lower FEV_1_ appears to be explained by FVC, suggesting disproportionate lung and airway growth that may contribute to asthma risk.^5^ While the growth beyond age 5 years was not assessed in this study, attaining or maintaining normal growth trajectories throughout childhood appears to be associated with improved lung function^7^ and could modify asthma risk or remission. Additionally, while we previously observed no overall microbiome differences by asthma status,^27^ altered gut microbiome profiles among children born preterm may still play a role in asthma risk,^28^ although the effect of postnatal microbiome modification remains uncertain.

Several limitations should be acknowledged. Asthma onset was not assessed, and hence causal association cannot be indicated. However, asthma in this population has likely developed before 9 years of age, suggesting relatively early‐onset asthma. Information on asthma phenotypes and endotypes was limited, and future studies are needed to examine whether specific growth trajectories are associated with distinct asthma phenotypes or endotypes. The lack of lung function measurements limited our ability to investigate potential mechanisms underlying the associations. Small for gestational age (SGA) status was not included; however, when applying the standard growth reference,^29^ the proportion classified as SGA was very low (<4%). Information on factors underlying preterm birth, beyond twin gestation, was unavailable. Moreover, the findings may be less applicable to prematurity driven by underlying pathological causes. The lower weight and height observed during the first months of life may reflect characteristics of early growth in twins, whereas growth thereafter appears comparable to that of singleton populations.^14^ The impact on the generalizability of the observed associations to singleton populations may be modest, as differences in childhood asthma risk between twins and singletons appear to be largely explained by gestational age and birth weight,^11^ which were accounted for in the analyses.

## 5 CONCLUSION

In this nationwide twin cohort, differences in average postnatal growth between children classified according to asthma prevalence at age 9 or 12 years were observed during the first 6 months of life; children with asthma had lower weight (−0.16 SDS) and height (−0.27 SDS). Thereafter, up to 5 years of age, the average growth pattern appeared similar in children with and without asthma. These early differences in postnatal growth were mainly seen among children born preterm. Trajectory analyses further suggested that increased risk of asthma was particularly observed in children with low birth size followed by catch‐up growth.

## AUTHOR CONTRIBUTIONS

**Resthie R. Putri:** Conceptualization; investigation; funding acquisition; writing – original draft; methodology; writing – review and editing; visualization; software; formal analysis. **Cecilia Lundholm:** Conceptualization; investigation; methodology; writing – review and editing; software; data curation; validation. **Mwenya Mubanga:** Writing – review and editing; investigation; validation; software; conceptualization; methodology. **Christian Trillkott:** Investigation; validation; writing – review and editing; software; conceptualization; methodology. **Vilhelmina Ullemar:** Investigation; writing – review and editing; software; data curation; validation; conceptualization; methodology. **Lars Sävendahl:** Conceptualization; investigation; writing – review and editing; methodology; validation. **Catarina Almqvist:** Conceptualization; investigation; funding acquisition; writing – review and editing; methodology; validation; project administration; data curation; supervision; resources.

## FUNDING INFORMATION

This work was supported by the Swedish Research Council (grant number: 2023‐02327; 2024‐03265), the Swedish Heart‐Lung Foundation (grant number: 20240974), the Swedish Asthma and Allergy Association's Research Foundation project (grant number: 2024‐0010), the Freemason Foundation for Children's Welfare in Stockholm, the HRH Crown Princess Lovisa Society for Child Care (grant number: 2026‐198), the Foundation of Sällskapet Barnavård (grant number: 2026‐035). All funders had no role in the design and conduct of the study.

## CONFLICT OF INTEREST STATEMENT

All authors have no conflicts of interest to declare.

## Supporting information


**Table S1.** Description of data sources.
**Table S2.** The number of individuals contributing data in each year.
**Table S3.** Association between growth trajectory groups and asthma.
**Table S4.** Joint categories of preterm birth and growth trajectories.
**Figure S1.** Scatterplots illustrating descriptive weight and height SDS patterns over time.
**Figure S2.** Weight and height SDS from 0 to 71 months (unadjusted) by asthma and preterm birth.
**Figure S3.** Weight and height SDS from 0 to 71 months (unadjusted) by asthma and preterm birth in the subset with complete data of all covariates.
**Figure S4.** Weight and height SDS from 0 to 71 months (adjusted for all covariates, including maternal BMI) by asthma and preterm birth.

## Data Availability

The data used in this study are not publicly available due to Swedish data protection legislation. Access to the data may be granted following approval by the Swedish Ethical Review Authority and application to the respective data custodians, including the Swedish Twin Registry, the National Board of Health and Welfare, and Statistics Sweden.
