# Supplementary material for: Postnatal growth and childhood asthma in preterm and full‐term twins: A cohort study
Source: Pediatr Allergy Immunol. 2026 Sep 16;37(9):e70488. doi: 10.1111/pai.70488 (PMC13580976; doi:10.1111/pai.70488)
Supplement: Supplementary file 1 [file PAI-37-e70488-s001.docx]

# Table S1. Description of data sources

| Data source | Description |
| --- | --- |
| Child and Adolescent Twin Study in Sweden | Since 2004, the STR has continuously contacted parents (or other guardians) of Swedish‑born twin children around the time the twins turn 9 or 12 years of age. Information on asthma and other diseases was obtained through parental interviews (overall response rate 69%), while growth data were extracted from child health records based on nurse‑measured growth charts.^1^ |
| Medical Birth Register | The register includes around 98% of all births in Sweden. Obstetric history, diseases, smoking habits, and height are self-reported, while weight is measured. The overall quality of data in the register is very good.^2^ |
| The Longitudinal Integrated Database for Health Insurance and Labor Market Studies | Education data are available in this database for almost all individuals aged 25 to 64 years residing in Sweden. The accuracy of the variable “highest attained level of education” is estimated to be 85%.^3^ |
| National Patient Register | This register contains all diagnoses in inpatient (nationwide since 1987) and outpatient specialized care (since 2001). The register has good diagnostic accuracy for most diagnoses, with a median positive predictive value of 84%.^4^ |

**References**

1. Zagai U, Lichtenstein P, Pedersen NL, Magnusson PKE. The Swedish Twin Registry: Content and Management as a Research Infrastructure. Twin Research and Human Genetics. 2019;22(6):672-680. doi:10.1017/thg.2019.99
2. Cnattingius S, Källén K, Sandström A, et al. The Swedish medical birth register during five decades: documentation of the content and quality of the register. Eur J Epidemiol. 2023;38(1):109-120. doi:10.1007/s10654-022-00947-5
3. Ludvigsson JF, Svedberg P, Olén O, Bruze G, Neovius M. The longitudinal integrated database for health insurance and labour market studies (LISA) and its use in medical research. *Eur J Epidemiol*. 2019;34(4):423-437. doi:10.1007/s10654-019-00511-8
4. Everhov ÅH, Thomas Frisell ·, Osooli · Mehdi, et al. Diagnostic accuracy in the Swedish national patient register: a review including diagnoses in the outpatient register. *Eur J Epidemiol*. Published online 123AD. doi:10.1007/s10654-025-01221-0

# Figure S1. Scatterplots illustrating descriptive weight and height SDS patterns over time

# Table S2. The number of individuals contributing data in each year

| **Age (months)** | **Number of individuals** |
| --- | --- |
| 0 – 12 | 2482 |
| 13 – 24 | 1714 |
| 25 – 36 | 1016 |
| 37 – 48 | 863 |
| 49 – 60 | 1433 |
| 61 – 71 | 1559 |

# Figure S2. Weight and height SDS from 0 to 71 months (unadjusted) by asthma and preterm birth

Average growth from 0 to 71 months in children **(N = 3059)** with and without asthma was assessed using generalized estimating equations (GEE), specifying a Gaussian family and an identity link function. **Weight SDS** and **height SDS** were the dependent variables, separately, while asthma status as the main independent variable. An exchangeable correlation structure was applied. Within-twin correlation was accounted for. The unadjusted model included time in days (modelled using restricted cubic spline) and an interaction term between asthma and time.

# Figure S3. Weight and height SDS from 0 to 71 months (unadjusted) by asthma and preterm birth in the subset with complete data of all covariates

Average growth from 0 to 71 months in children **(N = 2534)** with and without asthma was assessed using generalized estimating equations (GEE), specifying a Gaussian family and an identity link function. **Weight SDS** and **height SDS** were the dependent variables, separately, while asthma status as the main independent variable. An exchangeable correlation structure was applied. Within-twin correlation was accounted for. The unadjusted model included time in days (modelled using restricted cubic spline) and an interaction term between asthma and time.

# Figure S4. Weight and height SDS from 0 to 71 months (adjusted for all covariates, including maternal BMI) by asthma and preterm birth

In full-term and preterm groups separately, average weight and height growth from birth to 71 months among children **(N = 2534)** with and without asthma using generalized estimating equations (GEE) with a Gaussian family and an identity link were performed. **Weight SDS** and **height SDS** were modeled as separate dependent variables, with asthma status as the primary independent variable. An exchangeable correlation structure was applied and accounted for within-twin correlation. The unadjusted model included age in days (modelled using a restricted cubic spline) and an interaction term between asthma status and age. The adjusted model additionally incorporated maternal age, maternal body mass index, smoking exposure during pregnancy, maternal asthma, parity, and parental education. The estimated means for the adjusted analyses were performed with all covariates at means (i.e., mother’s age at 30 years, maternal BMI: 23 kg/m^2^, no maternal asthma, no smoking, parental education at high school level, parity=2).

# Table S3. Association between growth trajectory groups and asthma

|  | N = 2154 | | N = 1793 | | |
| --- | --- | --- | --- | --- | --- |
|  | % asthma | Unadjusted OR | % asthma | Unadjusted OR* | Adjusted OR** |
| Trajectory group |  |  |  |  |  |
| 1 (the lowest) | 22.4% (36/161) | **2.13 (1.30 - 3.48)** | 20.8% (26/125) | **2.03 (1.15 - 3.57)** | **1.89 (1.07 - 3.34)** |
| 2 | 19.2% (117/492) | **1.76 (1.22 - 2.54)** | 18.0% (93/516) | **1.70 (1.14 - 2.53)** | **1.61 (1.07 - 2.43)** |
| 3 | 13.4% (124/926) | 1.14 (0.81 - 1.62) | 13.8% (104/751) | 1.24 (0.85 - 1.81) | 1.22 (0.83 - 1.80) |
| 4 | 11.9% (57/478) | Ref | 11.5% (46/401) | Ref | Ref |

*Unadjusted odds ratio in the subpopulation with complete data of all covariates

**Odds ratio was adjusted for maternal age, maternal asthma, parity, parental education, and maternal body mass index

# Table S4. Joint categories of preterm birth and growth trajectories

|  | Unadjusted OR (N = 2174) | Unadjusted OR (N = 1793)* | Adjusted OR (N = 1793)** |
| --- | --- | --- | --- |
| Preterm & group 1 | **2.16 (1.28 - 3.63)** | **2.07 (1.13 – 3.78)** | **1.95 (1.07 – 3.56)** |
| Preterm & group 2 | **2.15 (1.41 - 3.27)** | **2.24 (1.41 - 3.56)** | **2.12 (1.32 – 3.39)** |
| Preterm & group 3 | 1.13 (0.69 – 1.85) | 1.19 (0.68 - 2.08) | 1.12 (0.63 – 1.99) |
| Preterm & group 4 | 0.93 (0.42 - 2.07) | 0.96 (0.40 - 2.27) | 0.97 (0.41 – 2.32) |
| Full term & group 1 | 1.46 (0.31 – 6.90) | 1.53 (0.32 – 7.29) | 1.38 (0.28 – 6.79) |
| Full term & group 2 | 1.26 (0.76 – 2.06) | 1.06 (0.61 - 1.82) | 1.02 (0.58 – 1.79) |
| Full term & group 3 | 1.13 (0.77 - 1.66) | 1.25 (0.83 - 1.90) | 1.26 (0.83 – 1.92) |
| Full term & group 4 | Ref | Ref | Ref |

*Unadjusted odds ratio in the subpopulation with complete data of all covariates

**Odds ratio was adjusted for maternal age, maternal asthma, parity, parental education, and maternal body mass index
